# Implant Body Fracture Due to Fatigue in a Maxillary Implant-Retained Overdenture Treatment

**DOI:** 10.1155/crid/4064452

**Published:** 2025-03-18

**Authors:** Hakan Terzioğlu, Can Hakan Sarıkaya, Rıza Gürbüz, Lale Karaağaçlıoğlu

**Affiliations:** ^1^Department of Prosthodontics, Faculty of Dentistry, Ankara University, Ankara, Turkey; ^2^Department of Prosthodontics, Graduate School of Health Sciences, Ankara University, Ankara, Turkey; ^3^Metallurgical and Materials Engineering, METU, Ankara, Turkey; ^4^Department of Prosthodontics, Faculty of Dentistry, Lokman Hekim University, Ankara, Turkey

**Keywords:** fatigue, fracture, implant, micromovement, overdenture

## Abstract

The aim of this study was to determine the causes of fatigue and implant failures in an implant-retained overdenture case. One out of four implants portrayed inadequate osseointegration during healing abutment placement, and one was fractured from the middle to the apical third after 1 year of denture use. Various aspects, such as insufficient cooling while implant socket preparation, incorrect prosthodontic planning, and erroneous design of the denture, were evaluated. Macroscopic and microscopic inspections of the fractured implant body were assessed by scanning electron microscopy (SEM) and optical microscopy. Consequently, metallic fatigue along with secondary cracks and beach marks were evident. It was deduced that the failed and fractured implants depicted substandard osseointegration; thus, they were susceptible to the wear out effect of the occlusal forces. Unfavorable osseointegration caused the fulcrum axis to move apically instead of forming on the neck part of the implant, and micromovements around the fulcrum axis mustered the fracture.

## 1. Introduction

The Lekholm and Zarb bone classification divides radiographic bone density into four groups in accordance with the amount of trabecular and cortical content [1]. From Type 1 to Type 4, not only does the compactness of bone starts to decrease but also the low-density trabecular bone starts to appear as the main scaffold. Vice versa, patients with Type 1 bone present marble-like features, meaning excessively condensed cortical bone with hardly any trabecular substance. Considering this phenomenon, patients who have undergone dental implant surgery having Type 1 bone sometimes require special treatments during the surgery to achieve prevailing osseointegration of the titanium implants [2, 3]. Thus, the variety of surface treatments and forms of alloys used as implant body materials are critical elements in establishing successful outcomes in both surgical and prosthodontic aspects [4]. One of this research's aims is to investigate the fatigue phenomenon in a soft tissue-supported implant-retained overdenture, since the literature can be considered lacking in such terms.

Dental endosseous implants in combination with implant-retained overdentures have become a popular alternative approach over the last 30 years when it comes to treating edentulous patients [5]. Consideration of implant-retained prostheses is mostly needed to increase patient comfort and masticatory function in comparison to conventional complete dentures by means of increased retention and stability along with reducing soft tissue dependence [5, 6]. Lots of studies show more than 90% success rates for mandibular overdentures [7, 8]; however, the success of maxillary overdentures mostly depends on the inspecting time and the clinician [7]. Implant-retained overdentures take advantage of both the mucosa and the implants, thus can be utilized where retention of the denture seems problematic. Some authors believe that the placement of ≥ 2 implants is considered adequate [9, 10]; some indicates minimum of four supporting implants with a palateless overdenture design, while others recommend ≤ 4 implants and palatal coverage [10]. Primary load-bearing areas apart from implants, such as tuber maxilla, buccal walls of alveolar bone, and palate, are fundamental components for the purpose of well-balanced load distribution [9]. Scarce quality with these parameters causes implants to sustain under higher stresses [11]; likewise, if the implant number, total implant diameter or the overall span of the implants are lower than advisable, palatal coverage and soft tissue management start to become critical for the overdenture planning. Therefore, appropriate extension of implant–overdenture basis becomes mandatory even though the patients' preference is palatally reduced design [10, 12].

## 2. Clinical Report

A 75-year-old male patient, with no reported systemic disorders or continuous medication use, presented with complete edentulism of both the maxilla and mandible, accompanied by severe bone resorption requiring clinical intervention. The patient's medical anamnesis revealed no significant psychosocial or hereditary conditions. Dental history assessment indicated that implant-supported prosthetic rehabilitation was recommended due to insufficient retention and stability of a conventional complete denture. The patient reported having used removable complete dentures for over 20 years with satisfactory functional and esthetic outcomes; however, increasing prosthesis instability and discomfort had progressively impacted his quality of life. Intraoral and radiographic evaluations revealed extensive alveolar bone resorption in the maxilla, with a marked reduction in trabecular bone structure and increased cortical bone density. Cone-beam computed tomography (CBCT) imaging confirmed significant resorption of the tuberosity region bilaterally, along with loss of crestal alveolar bone, findings attributed to long-term edentulism and advanced age-related bone remodeling. Given the compromised bone volume, a treatment approach involving an implant-retained overdenture with unsplinted ball attachments was planned for the anterior maxillary region, which exhibited the most favorable bone quality as determined by CBCT analysis. Four Zimmer dental implants were placed according to the FDI numeric classification: two in the 2.2 and 2.4 positions and two in the 1.2 and 1.4 positions. At the 6-month postoperative evaluation, the patient was scheduled for prosthodontic loading. However, implant failure was observed at Site 2.4 during the placement of the healing abutment, attributed to insufficient osseointegration. The failure was characterized by implant mobility and rotational instability upon abutment connection, necessitating reassessment of the treatment plan for that site.

**Figure 1:**
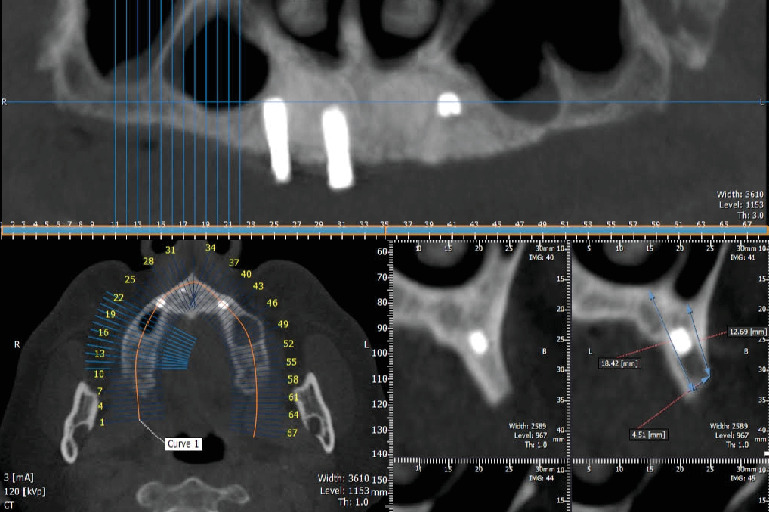
OPG and CBCT images reveal the fracture line of the implant after a month of use. Cross sections were evaluated in order to comprehend the exact position of the fracture line.

To ensure a cautious approach, the ISQ values of Implants 1.4, 1.2, and 2.2 were assessed, measuring 71, 73, and 69, respectively. The patient was advised to undergo bone augmentation and reimplantation procedures to compensate for implant loss; however, they were declined due to a lack of cooperation. As an alternative, the solidarization of the remaining implants through bar framework retention was proposed, but the patient was unwilling to cover the additional manufacturing costs. Consequently, the denture was loaded onto the remaining three implants (1.4, 1.2, and 2.2), with necessary occlusal adjustments. A bilateral balanced occlusion was selected, and modifications were made to maintain clearance during incisal and lateral guidance. Follow-up examinations were conducted the day after loading, as well as at 1 week, 1, and 3 months postprocedure, during which all primary contacts were eliminated. After a year of use, the patient started experiencing slight pain and a disturbing feeling on the implant site. Radiographs and intraoral examinations indicated that Implant No. 2.2 was fractured from middle to apical third, and when the denture was examined, a locator-type anchor attached to the regarding implant was noticed inside the metal housing. One question was the whereabouts of the fracture, which is normally expected around the crestal bone, where the fulcrum to the lateral forces occurs [13]. Due to unfavorable primary stability, an extended lever arm creates an excessive load on the implant body, especially at the point where osseointegration is complete and the axis of fulcrum forms. During the radiographic examination, orthopantomography (OPG) and CBCT images revealed the fractured implant and the level of fracture on the left maxillary canine area (Figure 1). The first assumption was inadequate osseointegration of the implant and loading of the prosthesis without achieving primary stability. Inevitably, the momentum point relocated to the apical third of the implant rather than the neck area observed in stable implants with ISQ values from 57 to 82 [14].

For this patient, however, no issue was observed either on the crestal bone or the implant-abutment connection. The main purpose was to understand why this phenomenon of change of fulcrum eventuated and, in this respect, the fractured implant was sent to further examination.

**Figure 2:**
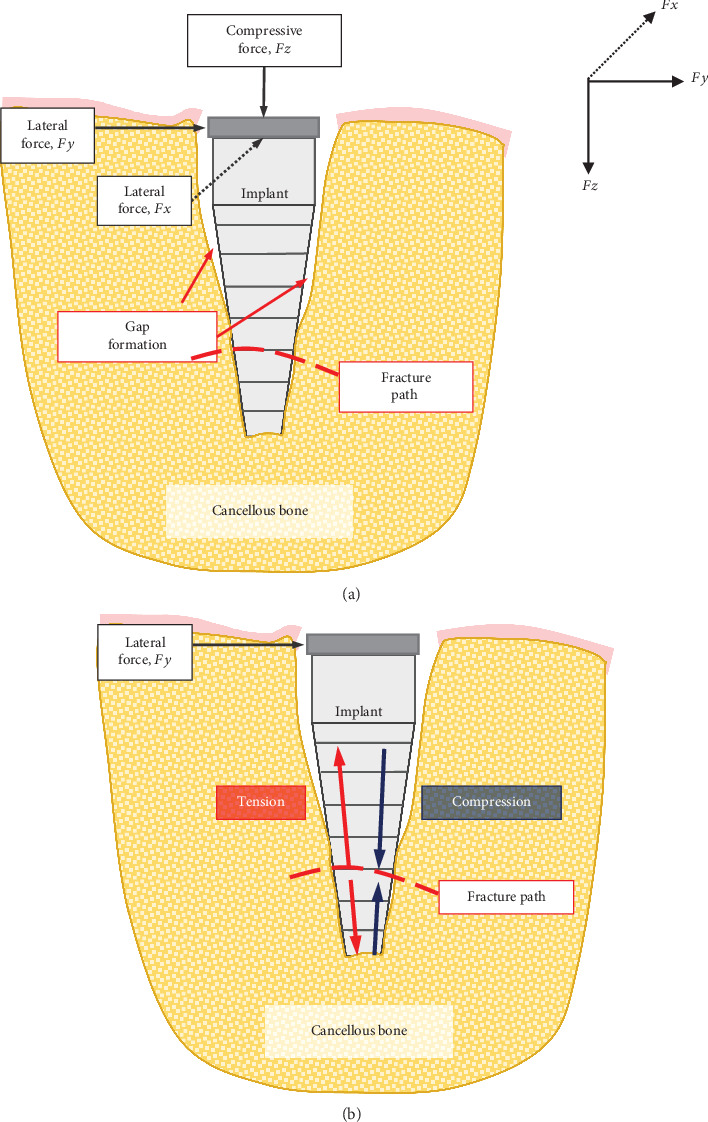
Lateral forces cause the formation of a bending moment at the fracture point. (a, b) Moment forms tensile stresses on one side of the implant and compressive stresses on the counter side. Repeated lateral forces during chewing action cause repeated tensile to zero fatigue loading cycles.

First, the fracture surface of the implant was examined macroscopically in order to determine the type of fracture and identify the marks which indicate fatigue failure (Figure 2a,b). After macroscopic investigation, the microstructure of the implant material was examined by using an optical microscope and scanning electron microscopy (SEM) to find metallurgical defects in the microstructure.

### 2.1. Stereoscopic Investigation of the Fracture Surface

**Figure 3:**
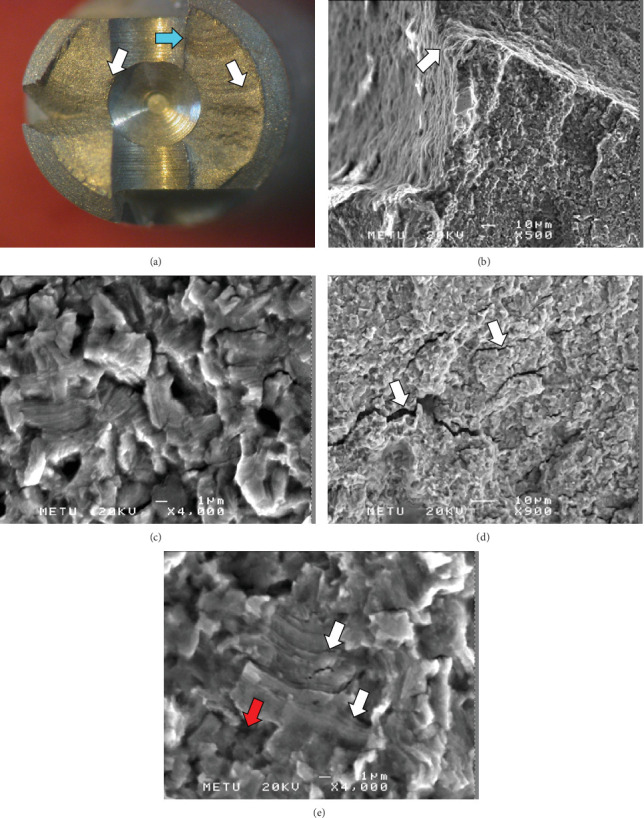
SEM results and images of the fractured implant body. (a) The crack initiation point (blue arrow) and beach marks (white arrows) indicating fatigue failure. (b) The point of initiation of the major fatigue crack. (c) Crack propagation region (in detail): Fatigue striations and secondary cracks are visible. The crack growth direction is downwards. (d) Crack propagation region, secondary cracks formed perpendicular to the crack growth direction. (e) The direction of crack propagation (red arrow) and fatigue striations (white arrows).

According to the results of macroscopic examination, neither metallurgical defect at the initiation point of the crack nor the heterogeneous structure on the stable oxide layer which forms during the anodizing process was detected. The initiation point of the crack and the beach marks which indicate fatigue failure are shown in Figure 3a.

### 2.2. SEM Examination of the Fracture Surface

As a result of the examination of the fracture surface via SEM, it was determined that the fracture occurred due to metallic fatigue. The initiation point of the crack is shown in Figure 3b. The crack propagation took place with the formation of fatigue striations and secondary cracks, and it is shown in Figure 3c,d. The direction of crack propagation can be clearly seen in Figure 3e.

### 2.3. Metallographic Investigation of the Implant Material

**Figure 4:**
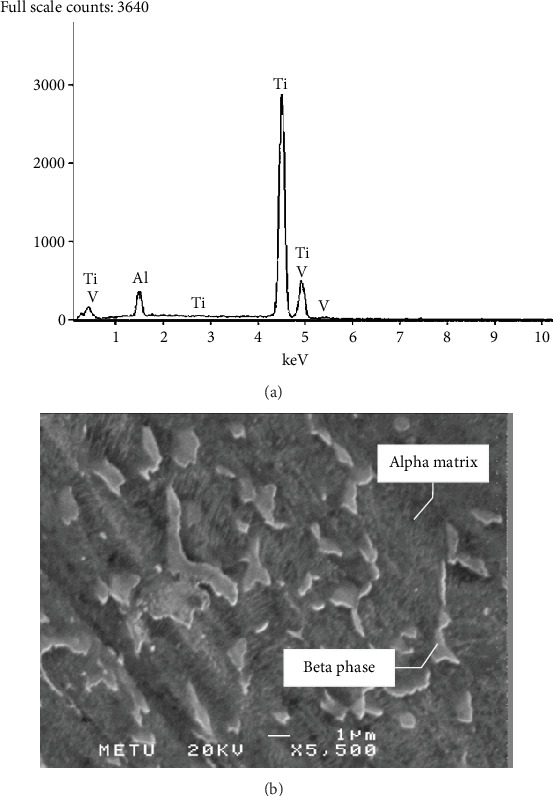
Microstructural evaluation of the fractured implant. (a) EDS analysis results which were obtained by using SEM. (b) SEM image taken from the surface of polished and etched metallographic specimen. The roughness in the surface is due to the attack of the etchant. It can be clearly observed that the microstructure consists of *α* matrix and *β* islands. The alloy is *α* + *β* alloy.

Energy-dispersive X-ray spectroscopy (EDS) and microstructure analyses were carried out using SEM. According to the EDS analysis results, it is determined that the implant material is Ti6Al4V alloy, and precipitation-hardened microstructure was observed during the microstructure analysis (Figure 4a,b; Table 1).

## 3. Discussion

For maintaining overdenture on implants, either splinted attachments like bars or unsplinted attachments such as stud attachments, telescopic attachments, ball anchors, and magnets can be exploited [11, 15, 16]. Locator attachments are preferred, especially because of their superiority over ball attachments in terms of less vertical space they require. Furthermore, these attachments are proved to be resilient and self-aligning, which can be adjusted up to 40° of angulation [17] and have dual retention with assorted color and retention value options [15, 18, 19]. However, inaccurate evaluation of vertical space examination or lack of uniform stress distribution in both peri-implant bone and prosthetic parts may lead to fractures or overcontour on prostheses, parting of attachments from the dentures, excessive vertical dimensions, along with patient dissatisfaction, and further loss of implants [18]. In order to prevent these problems, dentists are advised to proceed with detailed planning prior to treatment. Even though locator attachments are proved to offer successful overdenture mechanics for edentulous patients, there are several surgical pointers that will determine the overall treatment outcome as well.

The aim of this report is to discuss the etiological factors contributing to implant body fracture in a patient rehabilitated with an implant-retained overdenture in the maxilla and a complete denture in the opposing jaw. As the alveolar bone has low thermal conductivity, the irrigation of the burs during the preparation of the implant site has utmost importance since drilling without adequate irrigation may lead to overheating; ergo, necrosis of bone. Increased heat in the implant site causes the exchange of bone with fibrous tissue at the implant-bone interface, and this phenomenon can jeopardize the survival rate of the implants, especially after the load through dentures is applied [2, 3, 20]. Moreover, heat dissipation differs according to bone type and characterization [20, 21]. It was shown that the denser the bone, the more heat is generated, which concludes the fact that drilling on cortical bone will create more heat than that of medullar bone [22, 23]. Different cortical thicknesses are shown to affect temperature alterations during drilling, and with that in mind, the importance of irrigation starts to become vital for denser cortical bone structures in comparison to more trabecular bone structures [22]. According to authorities and publications, the temperature should remain under 47°C and the drilling time should be limited to 1 min [2].

Another parameter that needs to be considered is the material and the surface dynamics of the implant. In this study, surface characteristics of the fractured implant were examined macroscopically and microscopically to determine the reason for failure. As a result of the EDS analysis on SEM, the material of the fractured dental implant was found to be Ti6Al4V alloy, which is one of the most widespread and conventional alloys used as a dental implant material due to its biocompatibility, long-term stability, high strength, and different characteristic options through a variety of surface treatments [4, 24, 25]. There are three major parameters to assess dental and orthopedic implants: biocompatibility, mechanical compatibility, and morphologic compatibility [26–29]. If any of these parameters show incongruity, severe failures during or after implant-retained treatments will be observed.

Titanium and its alloys are commonly used in dental and orthopedic implants due to their low density, high strength, high corrosion resistance, long-term stability, and long fatigue life [30, 31]. In addition, the elastic modulus of titanium (110 GPa) is relatively closer to dense and low trabecular bone (1.47 and 0.231 GPa, respectively), as well as compact bone (14.7 GPa) [30], compared to other commonly used implant alloys such as chrome-cobalt alloys (218 GPa) [31]. Therefore, it has higher biocompatibility with human bone. Ti6Al4V and Ti6Al4Nb, which are also known as *α* + *β* alloys, have not only superior mechanical properties such as low fatigue cycle and corrosion resistance, but also high machinability and strength by means of ductile *α* phase and brittle *β* phase involvement in the microstructure [32, 33]. Supplementary alloying elements are entitled and addressed based on their effects on *α* and *β* phase formation. If alloying elements cause constant or increasing temperature to rise of *α* phase, these alloying elements are called “*α* stabilizer” such as aluminum, tin, and zirconium [34, 35]. Similarly, alloying elements that stabilize *β* phase are called “*β* stabilizer” such as vanadium and iron [35, 36]. Ti6Al4V is one of the most common *α* + *β* alloys which is formed by adjoining *α* stabilizers and a *β* stabilizer in the microstructure. The amount of *α* and *β* phases in the microstructure can change with respect to the amount and type of the *β* stabilizer [32–36].

There are three essential parameters to assess dental and orthopedic implants: biocompatibility, mechanical compatibility, and morphologic compatibility [29, 37]. Biocompatibility refers to the congruity and stability of the implant material with surrounding hard and soft tissues together. Mechanical compatibility means the surface resistance of the implant material against the external pressure of the patient's body. Implants should be placed properly to obtain homogeneous load distribution. Lastly, morphological compatibility stands for the integration between the surface of the implant material and neighboring bone [29, 37]. Dental implants are typically exposed to cyclic loadings which can cause metallic fatigue [38, 39]. Erroneously positioned implants lead to heterogeneous loads on the implants in addition to stress-raising effect of sharp edges on the macrostructure; thus, it decreases the fatigue life of the material [38–40].

The analysis done during this research establishes an important aspect for the implant-retained restorations and illustrates a significant perspective on this matter. Not only is soft tissue support highly encouraged for the implant-retained restorations, but also the clinicians should not take advantage of the reinforcement of the implants. EDS analysis result, which was realized using SEM, confirms that the material of fractured dental implant was Ti6Al4V alloy. During the examination of material's microstructure, precipitation hardened microstructure was detected, beta phase particles precipitated in the alpha matrix, and overall, the microstructure was observed to be homogenous, prompting long-term stability and increased fatigue life. Furthermore, in the heat treatment of the material, no defects were observed, and in the microstructure, there was not any casting defect nor casting cavity which induce fatigue failure.

Macro and micro level analyses of the fracture surface using optical microscope and SEM revealed beach marks, which were caused by interruptions in load cycles, changes in loading spectrum, or corrosion effects, and were taken as the indication of fatigue failure.

Although Ti6Al4V alloy is one of the most common implant body materials due to its biocompatibility and high resistance to fatigue [24], it was stated that fatigue failures might occur on the implant body due to stress-raising effect of the sharp edges. Since the load distribution on erroneously positioned implants is not homogeneous, stress-raising effect of the edge points increases and causes fatigue failure of the implant materials. It can be concluded that the load distribution on the fractured implant was not homogeneous due to the erroneous positioning of the implant, and mechanical compatibility could not be achieved. As a result of the heterogeneous distribution on the implant body, stress-raising effect of the sharp edges increased. Crack propagation developed with secondary cracks. As a result of the stress-raising effect of sharp edges on the implant, metallic failure occurred, and eventually, fracture developed.

In the light of these findings, failure and fracture of implant bodies and the heterogeneous stress distribution, which ultimately led to elevated stress levels, indicated insufficient osseointegration. With those in mind, our null hypothesis was that the fulcrum on the implant was not formed around the crestal bone level. The fracture line indicated that the micromovements which caused the implant to fracture were not on the implant-abutment connection, but on the implant itself. The bulk and heavy structure of the prosthesis, severe bone loss and unpromising soft tissue, insufficient number of implants, and the use of incorrect techniques during the prosthodontic building process were some of the other ideas of causality. Investigating the SEM results, the authors determined that the most probable phenomenon that occurred and resulted in the loss of two implants was the deficient levels of irrigation and cooling during the preparation of implant sockets since inadequate irrigation during drilling and the consequent excessive heat on the walls of the drilling socket jeopardize full healing and osseointegration [2, 3]. In a study done by Ahmadzadeh and Teimouri [41], three attachment types were investigated in terms of stress distribution. Among the four implants which were placed on the maxilla and similar placement to the case in this research, the highest amount of stress was observed in locator type attachments and around the cervical part of the implants on the working side, which is the second right premolar. The findings regarding the research were accepted, related, and deduced as supportive to the theory that the irrigation was under acceptable levels and therefore, implant failure, which is under the highest stress, was eventuated. Moreover, resorbed tubers on both sides of the maxilla and inadequate soft tissue support were disadvantageous and negative factors that eventually resulted in escalated stress on the implants' neck. Another theory developed and investigated at first was the effect of unfavorable prosthodontic design. However, as Ma et al. [42] and Kappel et al. [43] pointed out in their separate research, the prosthodontic design does not significantly affect the implant success rate for implant-retained overdentures loaded on three implants, and therefore, it was disregarded as well.

## 4. Conclusion

Endosseous dental implants have been widely utilized for the rehabilitation of patients with diverse treatment needs, often providing the most minimally invasive and conservative therapeutic approach. Similarly, implant-retained overdentures offer an effective solution for patients with significant alveolar bone resorption, enhancing comfort and functional stability compared to removable partial dentures. However, accurate diagnosis and meticulous treatment planning are critical to preventing complications that may compromise clinical outcomes. Essential factors for the success of implant-retained overdenture therapy include adherence to surgical protocols, precise implant site preparation following established drilling guidelines, presurgical prosthetic planning, and the selection of appropriate materials. In this study, our findings indicate that metallic fatigue, resulting from inadequate osseointegration and insufficient attachment between the implant and the surrounding bone, led to an altered fulcrum axis. This biomechanical imbalance induced micromovements, ultimately culminating in implant body fracture. Therefore, all surgical and prosthodontic considerations must be meticulously evaluated to ensure optimal clinical outcomes and long-term implant success.

## Figures and Tables

**Table 1:** Element composition table of EDS analysis results in Figure 3a.

**Element**	**Composition (in % by mass)**
Al	6.0
Ti	89.4
V	4.2

## Data Availability

Data sharing not applicable to this article as no datasets were generated or analyzed during the current study.
